# Saliva

**Published:** 1868-03

**Authors:** Andrew S. Cutler


### ARTICLE II.

### *Saliva.*

By Andrew S. Cutler, D.D.S.

This as a general term includes the secretions of the parotid, submaxillary, and sublingual glands. It is a transparent, viscid fluid, with but little taste or smell, when of a healthy recent condition. It is mixed also to a considerable extent with the mucus from the buccal, labial, and lingual glands. Its specific gravity according to Dalton, is 1.005. Different examiners, however, give different results, and it is a well ascertained fact that different pathological conditions give different degrees of density. Hamburger found it 1.0167. Lehman makes it from 1.004 to 1.006, and Golding Bird states it at 1.0091. Dr. Samuel Wright of Edinburg, Scotland, who has made this subject one of deep study and research, and whose opinion is entitled to profound deference, in a series of experiments upon the same individual, finds it of a higher specific gravity after a meal, than before, and in the evening, than early in the day. A free use of animal food also increases its density, especially when such food is of a fatty nature. A vegetable diet seems to materially lesson its specific gravity, while alcoholic stimulants again increase it. Dr. Wright found a difference according to the different varieties of food used, of from 1.0039 to 1.0176. Like most fluids of an adhesive nature, it becomes frothy when agitated, owing to the admixture of atmospheric air. It undoubtedly has a great affinity for oxygen, absorbing it readily from the atmosphere, and giving it out again to other bodies, hence it is, that silver is liable to tarnish or oxidize if left in contact with it for any considerable length of time. But this, it seems, may also be owing to the traces of sulphur discoverable in mucus. In the preparation of mercurial ointment, it is a well known fact that the globules of mercury are more easily broken down if there be an admixture of saliva from the mouth. All oxidizable metals also, seem to be more easily acted upon by this fluid than by pure water. Normal, healthy, saliva, according to Wright, under ordinary circumstances, absorbs about its own volume of oxygen, though under abnormal conditions it may absorb from one-half this amount to nearly twice as much. This is undoubtedly owing in part to the difference in proportion of the carbonic acid gas contained in the secretion. When of a recent state, saliva is frothy, and opaline in appearance, and holds in suspension, minute, whitish flocculi.

If allowed to stand for a sufficient length of time in a cylindrical vessel, there appears an opaque whitish deposit collecting at the bottom, the fluid above becoming clear· A microscopic examination of this precipitate, reveals an abundance of epithelium scales, probably detached from the internal surface of the mouth by mechanical irritation. There is also a certain amount of granular matter and a few oil globules to be detected. The organic substance of saliva is known as *ptyaline.* A principle analogous to *diastase*, a nitrogenous product. Both of these substances have the peculiar property of turning starch into sugar, and thereby induce fermentation. Ptyaline is coagulable by alcohol, which phenomenon is brought about by the small percentage of albumen it contains. Sulphocyanogen may also be detected by a solution of the chloride of iron, producing the red color of sulphocyanide of iron. Saliva has usually a decided alkaline reaction, which may vary in intensity according to circumstances. The amount of fluid secreted within a given time is also exceedingly variable, and may result from a great complication of causes, amongst which are the amount of fluids taken within the system, the character of these fluids, the amount of solid food ingested, its consistency, its digestive properties, the amount of motion of the muscles of mastication, and the particular physiological or pathological condition of the subject. Mitscherlisch obtained from a patient having a salivary fistula in the duct of steno, about two and one-half ounces of fluid in twenty-four hours. He calculated that the entire amount secreted was about six times that of this single gland. According to which calculation the patient must have secreted some fifteen ounces in the course of a day. Valentine placed the amount per 24 hours, at from seven to about ten ounces, whilst Thompson made a normal secretive amount at only seven and six-tenths ounces troy. These estimates without doubt fall considerably below a correct average. Bidder and Schmidt make much higher calculations. One of these observers in experimenting upon himself, without the use of any artificial stimula, and allowing seven hours for sleep, secreted nearly three and one-half pounds avoirdupois. Dalton under like conditions was able to collect about 556 grains of saliva per hour, and thinks that under ordinary circumstances the amount secreted may be rather less than three pounds avoirdupois per diem. But as before stated, owing to a great complication of causes, the normal flow of this peculiar fluid may very widely vary, it being necessary to take in consideration the size, sex, age, weight, food, drink, and particular pathological condition of the individual experimented on. In common with nitrogenous substances generally, saliva is susceptible to quick decomposition. This property is undoubtedly owing to the presence of its organic principle, ptyaline. When exposed to a temperature of 60° F. putrefaction may commence in four or five days, generating ammonia. About the year 1831, Leuchs of Germany, made known the remarkable fact, that if saliva were mixed with boiled starch in equal proportions, and kept for a short time at a temperature of 100° F. the latter was converted into sugar. According to Dr. Wright, it seems probable that the theory implied, was more or less understood for a long time prior to the experiments of that chemist. And though Mr. Leuchs has generally the credit of making the discovery, one thing is certain, the principle involved was certainly made use of many years before his time. The transformation of starch into sugar, is undoubtedly one of catalysis, or simple contact of the starch with the organic substance of the saliva. This peculiar phenomenon was for a long time supposed to constitute the physiological function of this secretion, viz. to effect the division and liquefaction of all starch principles. This property, however, seems an accidental one, for under the ordinary processes of digestion, the food does not remain for a sufficient length of time in contact with the fluids of the mouth, to be acted upon by them, for, passing almost directly into the stomach, it becomes rapidly intermingled with the fluids of that organ, and when there, this peculiar chemical action of saliva upon all starch material, seems at least for the most part, at an end. For if starch, saliva, and gastric juice be mixed together in a test tube, no change, indicating the presence of sugar takes place. In fact, there seems to be an altogether distinct provision made for the digestion of this substance, entirely independent of the action of saliva. And that provision is met in the smaller intestines and their secretions. Here the conversion of the starch group into the saccharine fluid, seems rapid, effective, and complete. The action in this case being far more distinct and energetic, than in the case of saliva. Still, it seems probable that saliva may assist, to a greater or less degree, in the various nutritive and digestive processes. Liebig thought that one great function of this fluid was to convey atmospheric air, particularly its oxygen into the stomach and intestines. And when we consider that a certain amount of oxygen seems necessary in these organs, for the accomplishing of their particular physiological offices, the above hypothesis seems not wholly improbable. Still, there is little doubt at the present day but that the great function of saliva is chiefly physical in its nature. Yet notwithstanding this, it is none the less essential to the actual necessities of the system. By its action the food is moistened, the process of mastication facilitated, and the mass intermingled and enveloped by a glairy viscous fluid, necessary for its lubrication in its downward passage through the æsophagus. Were it not for this fluid of the mouth, all dry food would have to be moistened by some unnatural vehicle, and an inflamed condition of surrounding parts, would naturally result. In fact, all food, whether containing a greater or less amount of moisture, seems to require, at least to some extent, the presence of the combined salivary secretions. And the fact we believe seems well ascertained, that where this deficiency is noted, there is also an accompanying, unfavorable, pathological condition. Whether this condition be the result of a general sympathy of parts, or of a direct action, or want of action of the substance in question, we will not undertake to determine. The chemical composition of saliva according to Bidder and Schmidt, is as follows:

| | |
|---|---|
| Water | 995.16 |
| Organic matter or ptyaline | 1.34 |
| Sulphocyanide of potassium | .06 |
| Phosphates of soda, lime and magnesia | .98 |
| Chlorides of sodium and potassium | .84 |
| Mixture of epithelium | 1.62 |
| | 1000.00 |

The above proportions, however, may vary in different individuals, and to some extent, even under normal conditions of the same individual. Berzelius found a greater proportion of animal matter.

| | |
|---|---|
| Water | 992.9 |
| Ptyaline | 2.9 |
| Mucus | 1.4 |
| Extract of flesh with alkaline lactates | .9 |
| Chloride of Sodium | 1.7 |
| Soda | .2 |
| | 1000.0 |

Wright found a greater proportion of water than either of the foregoing. While Frerichs, quoted by Carpenter, the great English physiologist, effects a sort of compromise with the preceding tables. It is as follows:

| | |
|---|---|
| Water | 994.10 |
| Ptyaline, with a little alcohol extract | 1.41 |
| Mucus and epithelium | 2.13 |
| Fatty matter | .07 |
| Sulphocyanide of potassium | .10 |
| Alkaline and earthy chlorides and phosphates with oxide of iron | 2.19 |
| | 1000.00 |

We now come to a brief consideration of the individual secretions entering as components of this fluid. First in importance, and greatest in amount, is the secretion of the parotid glands. This fluid may be obtained in a pure state by inserting a silver canula of a diameter of from 1.25 to 1.20 of an inch, into the orifice of the duct of Steno. Human parotid saliva is colorless, perfectly clear, watery in consistency, and distinctly alkaline in reaction. When the jaws are in a state of rest its flow is scanty. But with the introduction of food into the mouth, in connection with the movements of the muscles of mastication, it is poured out in considerable quantity. Dalton obtained under favorable circumstances, from a single duct of a healthy man, 480 grains of parotid saliva in twenty minutes. This fluid was analyzed by Perkins, of the College of Physicians and Surgeons, of the city of New York. The following were the results:

| | |
|---|---|
| Water | 983.300 |
| Organic matter precipitable by alcohol | 7.352 |
| Substances destructible by heat, not precipitable by alcohol | 4.81 |
| Sulphocyanide of sodium | .33 |
| Phosphate of lime | .24 |
| Chloride of potassium | .90 |
| Chloride of sodium and carbonate of soda | 3.06 |
| | 1000.00 |

Organic matter, or ptyaline seems to enter into a greater proportion here, than it does in the mixed saliva. The flow of this fluid presents one peculiarity in the fact that it is poured out most freely upon the particular side in which mastication is taking place at the time. The secretion of the sub-maxillary glands is less in quantity, and more difficult to obtain in a pure state than the parotid. Its specific gravity is rather less than that of the mixed secretions, nor is its alkaline reaction so decided.

While the amount of organic matter contained in it, is somewhat less than is to be found in either the combined or parotid fluids. There is, however, a noted increase in its viscidity. The characteristics of sublingual saliva are not so well understood as either the individual fluids before mentioned. They are, undoubtedly, however much the same as those of the submaxillary. There being, possibly, a still increased degree of viscidity, with a smaller proportion of organic elements.

### Abnormal Conditions of Saliva.

Of the chemico pathological conditions of combined saliva, we will devote only the briefest time and space. The following classification is adopted from Dr. Samuel Wright.

Deficient, Redundant, Fatty, Sweet, Albuminous, Bilious, Bloody, Acid, Alkaline, Calcareous, Saline, Puriform, Fetid, Acrid, Colored, Frothy, Gelatinous, and Milky.

Deficient Saliva. This variety arises from a cause purely physiological in its nature, and may be owing to peculiar nervous or emotional conditions. Or, it may arise from an obstruction of the salivary ducts, or an inactivity of the secreting glands. An obliteration of the passage leading from the glands, gives rise to the disease termed Ranula, which is merely a distension of the duct on account of the closure of its natural outlet. Besanez found an analysis of ranula fluid like the following.

| | |
|---|---|
| Water | 95.029 |
| Traces of fat and chloride of sodium | 1.062 |
| Watery extractive matters | .923 |
| Albuminate of soda | 2.986 |
| | 100.000 |

Redundant Saliva. This variety may be either spontaneous and healthy, or excited, with a vitiated and altered condition of its constituents. The former is frequently, and the latter occasionally met with in young children during the "teething period." Its specific gravity in such cases is lower than that found in the normal flow of the secretion being from 1.001 to 1.003 or 1.005. This super-abundance of saliva is often observable in extreme old age, and is frequently noticeable under many conditions of idiocy. The fluid is usually clear and transparent, without the usual blue tinge of the normal secretion. It is often deficient in albumen, with a corresponding small percentage of ptyaline and sulphocyanogen. From an examination of spontaneous salivation, Vogel makes the following table.

| | |
|---|---|
| Water | 991.2 |
| Ptyaline, osmazone, fat and albumen | 4.4 |
| Salts of soda, potash and lime | 4.4 |
| | 1000.0 |

Artificial ptyalism, or excessive discharge of saliva, is, in many parts of our country, the too common result of an injudicious or excessive use of mercurial preparations. It is also occasionally brought about by an undue use of the halogens, especially Iodine, Chlorine, and Bromine. Arsenic, Lead, and antimony frequently effect the same results. When ptyalism is produced by mercurial agency, the quantity of fluid discharged, its specific gravity, and chemical components are all liable to considerable variation. Lehmann thinks that in this condition of disease, the traces of the metal itself are clear and distinct. Other noted physiological chemists have never been able to detect its presence. The specific gravity of salivated fluid is much the same as in cases of spontaneous ptyalism. There may be, also, a noted excess of mucus, indicating a higher specific gravity, usually with a loss of its peculiar transparency, unless the mucus be in great excess, when the turbidity frequently vanishes. The alkaline reaction, too is unmistakeable and its property for absorbing oxygen increased. There is often, also, an increased amount of ptyaline present, effecting an easy decomposition, and generating ammonia. If the excess of ptyaline be very noted, there is usually a deficiency of sulphocyanogen. In giving an analysis of mercurial ptyalism, we can probably do no better than quote the result of three analyses of the distinguished Dr. Wright.

| | No. 1 | No. 2 | No. 3 |
|---|---|---|---|
| Water | 989.8 | 988.7 | 987.4 |
| Ptyaline | 1.7 | 1.9 | 2.7 |
| Fatty acid | trace. | .4 | .7 |
| Albumen with soda and albuminate of soda | 1.5 | .6 | 1.3 |
| Mucus with trace of ptyaline | 2.1 | 3.8 | 3.5 |
| Lactates. Muriates. Phosphates. { Potash. Soda. Lime. } | 1.9 | 2.4 | 2.6 |
| Hydrosulphocyanites | 3.0 | 2.2 | 1.8 |
| | 1000.0 | 1000.0 | 1000.0 |

Fatty saliva. There may be, as has before been intimated, under normal conditions, a certain proportion of fatty matters in the saliva, where these are exhibited in undue amount, the peculiar condition under consideration is presented. Fatty saliva is characterized by a greasy taste and feeling in the mouth accompanied by a sticky, slimy sensation. Its specific gravity is often as great as 1.0113. Its color is a dull yellowish white, without the usual blue tinge of the healthy state. Its property of absorbing oxygen is limited, and its action upon starch, feeble, this substance being imperfectly converted into gum, with no traces of sugar. Wright made an analysis like the following.

| | |
|---|---|
| Water | 987.4 |
| Ptyaline | .7 |
| Fatty matter and acid | 3.9 |
| Albumen and soda with albuminate of soda | 1.5 |
| Sulphocyanide of potassium | trace. |
| Mucus | 2.4 |
| Lactates, muriates and phosphates of potash, soda and lime | 1.8 |
| Loss | 2.3 |
| | 1000.0 |

The true organic principle is here notedly deficient, and, as is usual with many abnormal conditions of this secretion, the proportion of water is in much less than normal quantity.

Sweet Saliva. This is a result of various abnormal conditions of the system. A depraved digestion, being, perhaps a predominating cause. It can be distinguished by a sweet, and often sickish taste, with a color nearly white, lifeless and lustreless. It decomposes readily, forming, as might be expected, acetic acid. Its chief peculiarity consists in the presence of saccharine matter in combination with mucus. Wright finds a table of its chemical elements as follows.

| | |
|---|---|
| Water | 986.9 |
| Ptyaline | .3 |
| Fatty acid | .2 |
| Muco saccharine matter | 5.6 |
| Albumen with soda and albuminate of soda | .4 |
| Mucus with trace of ptyaline | 2.6 |
| Lactates, muriates and phosphates of soda potash and lime | 1.9 |
| Loss | 2.1 |
| | 1000.0 |

Albuminous Saliva. Of this description there are two general varieties. The transparent, and the opaque. The former contains less ptyaline and more sulphocyanogen than the healthy secretion. Its specific gravity and alkalinity is also much increased. It is easily decomposed, and as is usual where there is organic matter in more than normal proportions, a larger percentage of ammonia is generated. Its action upon starch is not so definite as in the case of the natural secretion. The opaque variety has a high specific gravity, is milky in appearance, and when boiled coagulates in flakes. These, in time are precipitated, leaving above, a greenish, semi-transparent fluid. The chemical action of this variety is strongly alkaline, absorbing but little oxygen, and exhibiting but faint powers over starch substances. This condition of disease is often found in persons addicted to an excessive use of alcoholic drinks, and also to inordinate eaters. As might be supposed, Albuminous Saliva may be distinguished by the increased percentage of albumen it contains. Any proportion either way of .02 minimum, or 5 per cent maximum constitutes diseased action. Wright, in one case found it as high as 1.03.

Bilious Saliva. When the action of the liver is abnormal, and its peculiar secretion in excess, or the secreting surfaces disordered, the bile becomes absorbed by the system in general, giving to all the tissues that peculiar color and expression, of which the term, "bilious," is, perhaps, more expressive than any other. This characteristic extends to the saliva, as well as to the other constituents of the body. Bilious saliva may be either colored or not. The lighter shades of this abnormal condition of the secretions of the mouth are usually alkaline, while the darker varieties may be acid. Bilious saliva is characterized by a high specific gravity, and a maukish, bitter, offensive taste. Both colored and colorless varieties contain but remote traces of ptyaline and sulphocyanogen, while their property of converting starch into sugar is almost entirely wanting. Their distinguishing peculiarity is owing to the presence of some 3.6 parts per 1000, of biliary matter and cholerestine.

Bloody Saliva. This variety may arise from different pathological causes. Hæmatin, the coloring principle of blood, giving the various degrees of shade, according to the amount of its presence, or the diseased action of parts with which it has come in contact. It has a high specific gravity, the usual percentage of sulphocyanogen, with a noted decrease however, in the presence of ptyaline. Oxygen is but slightly absorbed by it, and its digestive powers are small. By decomposition it becomes darker and darker, evolving ammonia.

Acid Saliva. It is this particular condition of the fluids of the mouth that has, perhaps, a more especial bearing upon the dental profession, than any of the others. The presence of the acids in the mouth, even when in a state of remote dilution, is productive, to a greater or less extent, of caries of the teeth, dissolving as they inevitably must, the calcarerous salts of these organs, rendering them porous and friable, and giving to them, also, an abnormal sensitiveness to every varying degree of heat and cold, and often even to touch itself. The peculiar sensation of "teeth upon edge," is undoubtedly due to the presence of acid in some form within the organic structures. This morbid state of the salivary secretions may be owing to different causes. Primarily and strictly local of which is a peculiarly abnormal condition of the glands themselves. Or, there may be, so to speak, an acid diathesis of the system generally, and this condition is really implied in different degrees in such diseases as rheumatism, gout, ague, various kinds of fever, and some particular functional disturbances of the stomach, and whenever, in fact, the saliva becomes acid to any considerable extent, this latter pathological state of the system is its sure accompaniment. The saliva, according to Wright, may be impregnated with lactic, acetic, muriatic, uric, or oxalic acids. Each particular variety being indicative, as a general thing, of some diseased action of particular parts. Acidity of the saliva is sometimes so excessive as to corrode even the gums and lips, while its affinity for the mineral substances of the teeth is so great, and its action so rapid, as to cause a disintegration of their structure, leaving them sensibly rough both to touch and appearance. The chemical test for the presence of acid, is litmus paper, which is colored with an intensity in proportion to the presence of the acidifying principle. Acid saliva has nearly the same specific gravity as the normal secretion and also the usual percentage of organic matter, which often presents distinct whitish floculi, the result of the coagulation of albumen from the action of the contained acid.

Alkaline Saliva. This variety is dependent upon an ex-excess of some alkaline principle within the system. It is generally found in connection with neuralgic or nervous affections particularly upon the diseased side. It does not possess the ready action upon starch as does healthy saliva, and is susceptible to a more rapid decomposition. Litmus paper gives no notice of the presence of acid, as all such properties have been neutralized by an excess of alkali, while its taste is sometimes so excessively characteristic of the above principle, as to lead one to suppose that free soda had accidentally found its way into the mouth. The specific gravity of this description of saliva is usually low, and one of its peculiar features is, that soda is the sole alkaline principle, all other alkalies being excreted by other sources.

Calcareous Saliva. Owes its peculiarity to the presence of the phosphates and carbonates of lime, and it becomes a matter of considerable interest to the dental practitioner from the fact that from it, chiefly arise those calcareous deposits found upon the teeth and within the salivary ducts In one case Dr. Wright found as high as 1.4 per cent of phosphate of lime in the saliva, the normal proportion being only about 16 parts per 1000. The other chemical elements may be in usual amount.

The remaining morbid conditions of saliva are either comparatively rare in occurrence, or unimportant in their nature. Their names are characteristics of their chemical or physical properties. Saline saliva implies the presence of a limited proportion of chloride of sodium. Puriform saliva always contains an admixture of pus, and characterizes the salivary fluids during the suppurative process of alveolar abscess and other diseased conditions of the gums. Its very nature implies an increased amount of albumen, and a noted susceptibility to decomposition. Fetid saliva generally considered, may owe its properties to the absorption by the saliva glands, of any nauseous or disagreeably scented substances taken within the system, and then giving out again their particular odor. A common and very disagreeable illustration of which is furnished by chewers of tobacco. Pathologically considered, however, fetid saliva is dependent upon morbid constitutional changes, which it is true, may to a greater or less extent be brought about by these same general or external causes. Acid saliva, without any essential changes of chemical ingredients or any discernable alteration of parts, may possess active chemical, or chemico-vital properties, materially differing from the healthy secretion. It is peculiarly manifest, when from an internal diseased origin, or external irritating cause, the normal action of the brain becomes excessive, and the animal or individual becomes so to speak infuriated. This property of saliva is noticable in hydrophobia, and also in many instances where animals are under the influence of uncontrollable frenzy. The foregoing morbid conditions of the salivary fluids, excepting, perhaps the last, are, it must be remembered, the results of diseased action, and not their primary cause. They may afford however, to the skilful pathologist, valuable indications of abnormal conditions of the system, whereby he may intelligently judge, or may be assisted in judging, at least to some degree, of the proper methods of controlling disease. As a general rule, we find in all inflammatory diseases, a noted decrease of the watery proportion of saliva. Below is given a comparative table of the general constituents of the salivary secretions in an average of ten analyses, from patients suffering from inflammatory action, and also when in a state of health. It is from L'Heritier.

| | In Inflammation. | In Health. |
|---|---|---|
| Water............ | 968.9 | 986.5 |
| Organic Matter | 30.0 | 12.6 |
| Inorganic Matter | 1.1 | .9 |
| | 1000.0 | 1000.0 |

